# Deep brain–machine interfaces: sensing and modulating the human deep brain

**DOI:** 10.1093/nsr/nwac212

**Published:** 2022-10-07

**Authors:** Yanan Sui, Huiling Yu, Chen Zhang, Yue Chen, Changqing Jiang, Luming Li

**Affiliations:** National Engineering Research Center of Neuromodulation, Tsinghua University, Beijing 100084, China; National Engineering Research Center of Neuromodulation, Tsinghua University, Beijing 100084, China; National Engineering Research Center of Neuromodulation, Tsinghua University, Beijing 100084, China; National Engineering Research Center of Neuromodulation, Tsinghua University, Beijing 100084, China; National Engineering Research Center of Neuromodulation, Tsinghua University, Beijing 100084, China; National Engineering Research Center of Neuromodulation, Tsinghua University, Beijing 100084, China; Precision Medicine & Healthcare Research Center, Tsinghua-Berkeley Shenzhen Institute, Tsinghua University, Shenzhen 518055, China; IDG/McGovern Institute for Brain Research at Tsinghua University, Beijing 100084, China; Institute of Epilepsy, Beijing Institute for Brain Disorders, Beijing 100069, China

**Keywords:** deep brain–machine interface, sensing and modulation, deep brain stimulation, stereotactic electroencephalography

## Abstract

Different from conventional brain–machine interfaces that focus more on decoding the cerebral cortex, deep brain–machine interfaces enable interactions between external machines and deep brain structures. They sense and modulate deep brain neural activities, aiming at function restoration, device control and therapeutic improvements. In this article, we provide an overview of multiple deep brain recording and stimulation techniques that can serve as deep brain–machine interfaces. We highlight two widely used interface technologies, namely deep brain stimulation and stereotactic electroencephalography, for technical trends, clinical applications and brain connectivity research. We discuss the potential to develop closed-loop deep brain–machine interfaces and achieve more effective and applicable systems for the treatment of neurological and psychiatric disorders.

## Teaser

Deep brain–machine interfaces bridge machines and deep brain structures essential to our vital functions, and provide promising treatments for neurological and psychiatric disorders.

## INTRODUCTION

**Figure 1.**
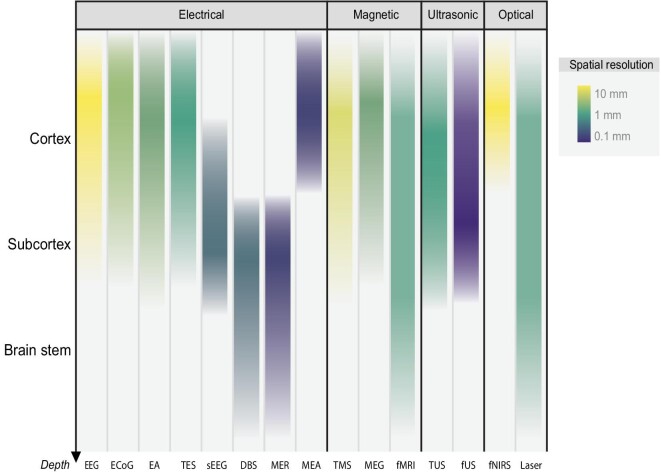
Common approaches for sensing and modulating the human brain with depth distribution and spatial resolution. The horizontal axis lists major electrical, magnetic, ultrasonic and optical approaches including EEG (electroencephalogram), ECoG (electrocorticography), EA (endovascular approach), TES (transcranial electrical stimulation), sEEG (stereotactic electroencephalography), DBS (deep brain stimulation), MER (microelectrode recording), MEA (microelectrode array), TMS (transcranial magnetic stimulation), MEG (magnetoencephalography), fMRI (functional magnetic resonance imaging), TUS (transcranial ultrasound), fUS (functional ultrasound), fNIRS (functional near-infrared spectroscopy) and laser therapy. The vertical axis represents the depth range covered by each approach from surface to deep brain. Color represents the scale of spatial resolution, with opacity illustrating the application rate of each approach at different depth levels. EEG, ECoG, MEA, MEG, fMRI, fUS and fNIRS are mainly used for sensing, whilst sometimes stimulation could also be delivered with ECoG and MEA electrodes. TES, TMS, TUS and laser therapy are mainly for modulation. EA, sEEG, DBS and MER can be used in both modalities.

Brain–machine interfaces (BMIs, also known as brain–computer interfaces) provide novel approaches for humans to interact with external devices and the environment. They help restore, improve and modulate human physical or mental functions [1,2]. Scientists investigate electrical, magnetic, ultrasonic, optical and other physical technologies to interface with the brain at different levels, as shown in Fig. 1. The cerebral cortex has long been the major target of brain–machine interface research. Investigators record and interpret neural activities of multiple cortical areas to understand human intentions, enable paralysed patients to control robotic arms and prostheses, and assist disabled people to communicate efficiently [3,4]. They also make efforts in setting up diagnostic procedures and treatments for brain injuries and neurological and psychiatric disorders through neural interventions [5–7].

While interfacing with the human cerebral cortex allows us to decode sensory and motor signals such as visual responses, hand movements and speech in labs [8,9], there is still a long way to go before these BMIs will be deployed in daily life or on a large scale. Subcortical areas (e.g. the substantia nigra, thalamus, hippocampus, etc.), contributing to various cognitive, affective, social and critical life functions, are the main targets for invasive neuromodulation research and clinical neurotherapeutics [10]. For treatments of many neurological and psychiatric disorders, interacting with deep brain structures is necessary and applicable.

**Figure 2.**
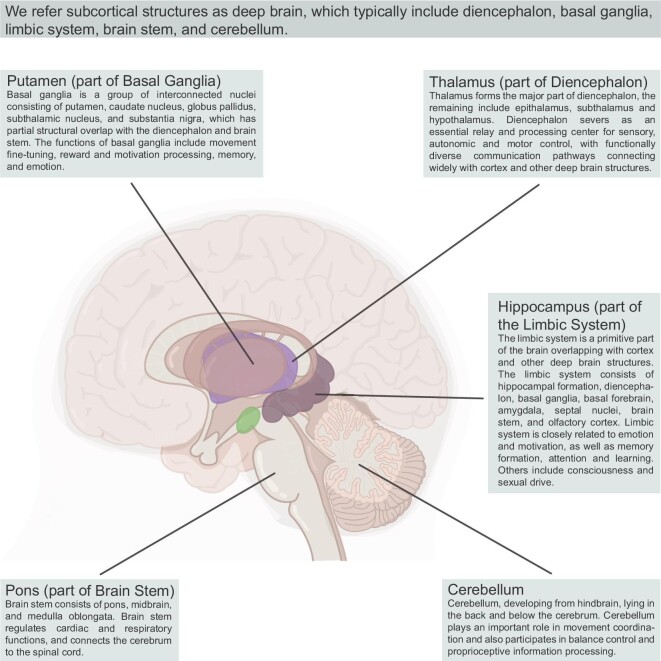
Deep brain structures and their main functions.

Deep brain structures (including basal ganglia, limbic system, diencephalon, cerebellum and brain stem) contribute to our vital functions ranging from sensory and motor to cognition and consciousness, as shown in Fig. 2. They are primitive and essential for our lives. Structural and functional abnormalities of deep brain structures are observed in multiple neurological and psychiatric disorders such as Parkinson's disease, Alzheimer's disease, depression, obsessive–compulsive disorder, etc. The Deep Brain Machine Interface (DBMI), focusing on the understanding and modulation of neural activities in deep brain structures, is an emerging research field with great application potential.

Besides recording and decoding, DBMIs are able to modulate deep structures and pathological states of the brain by delivering therapeutical stimulations. Advanced DBMI technologies aim at recording and decoding deep neural activities with high spatio-temporal resolution and effectively configuring stimulation parameters that can precisely regulate brain states. Due to our limited understanding of fundamental mechanisms and plasticity as well as adaptability of the central nervous system, the development of DBMIs with long-term efficacy is still challenging.

Since electrical signals recorded directly from brain tissues convey information of neuronal communication, electrical BMIs have attracted the most research interest in this field [11]. In this review, we first overview the current neural electrical activity-based BMI technologies and their applications in sensing and modulating deep brain structures. We then present two widely used DBMI systems, namely deep brain stimulation (DBS) and stereotactic electroencephalography (sEEG), with a special focus on the latest technical advances and current clinical applications. The potential of DBMIs to be used as powerful brain research platforms is addressed along with their therapeutic utility. We also discuss the closed-loop framework for DBMI systems, providing a perspective on technical and clinical developments of closed-loop DBMIs.

## FROM SURFACE TO DEEP BRAIN: THE NEW TREND IN BRAIN–MACHINE INTERFACES

Electrical activities in our brain underlie the coordination of the thoughts, emotions and behavior of humans. Neural electrical activity-based BMIs capture and/or modulate these brain dynamics directly. As our understanding of brain functions deepens, we are seeing a new trend in BMI research that expands interest and focus from interacting with the cortical areas of the brain to deep brain structures, regardless of the deployed interface technology.

**Figure 3.**
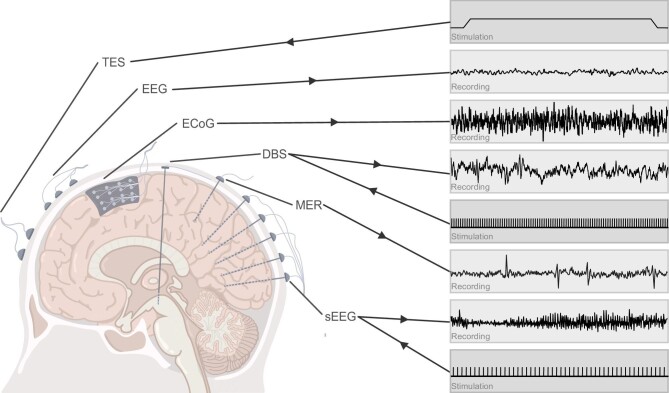
Major electrical recording and stimulation approaches for brain–machine interfaces. EEG, ECoG and MER for recording; TES for stimulation; DBS and sEEG for both recording and stimulation.

The main electrical sensing and modulating approaches for the human brain include electroencephalography (EEG), transcranial electrical stimulation (TES), electrocorticography (ECoG), microelectrode recording (MER), stereotactic electroencephalography (sEEG) and DBS. Figure 3 shows examples of non-invasive recordings with EEG and the non-invasive stimulation pattern of TES. ECoG is usually used for cortical recordings while MER is used mainly for subcortical recordings. DBS and sEEG are capable of both recording and stimulation in deep brain areas. We also show sample recordings and stimulation patterns of sEEG and DBS in Fig. 3.

EEG is a non-invasive way to sense the electrical activities traditionally from the surface of the brain with electrodes placed onto the scalp. EEG has been the most popular interfacing modality for BMI in the past decades. EEG-based BMIs have been adopted in cognitive and behavioral research and robotic controlling, assisting in the diagnosis of neurological and mental disorders and neurorehabilitation, with impressive cases of neuroprosthetic operation and communication restoration for disabled individuals [12–14]. Typical neural signals utilized in EEG-based BMI systems are evoked or event-related potentials and brain oscillations. More recently, EEG-based decoding approaches have aimed to leverage deep learning frameworks [15–17]. High-density recordings, novel signal-processing methods and innovative algorithms have helped to extend the potential applications of EEG-based BMIs. At the forefront of current development, several studies have shown evidence of the EEG-based reconstruction of neural source activity from subcortical structures [18,19], providing us with a possible approach for non-invasive deep brain recording.

While EEG is designed for recording, TES is a non-invasive technique for electrical brain stimulation delivered through the scalp to modulate functional connectivity and cortical excitability [20]. It is increasingly applied in treatments of various neurological and psychiatric disorders such as Alzheimer's disease, motor impairment after stroke, depression, etc. A recent study with epileptic patients who simultaneously underwent intracranial recordings showed that transcranial alternating current stimulation (one major modality of TES) delivered with small high-definition electrodes at low intensities could induce an electrical field in deep brain structures [21]. Efforts in stimulation waveform and pulse design, and optimization of electrode configuration might also facilitate DBS with TES according to animal and modeling studies [22]. These results demonstrate the possibility of modulating deep brain networks through non-invasive approaches. EEG and TES could also be combined as a closed-loop system for non-invasive clinical interventions [23].

Despite the relatively high temporal resolution of EEG and TES, low spatial resolution and attenuated signals are still the major limitations due to the resistances and filtering effects of cerebrospinal fluid, dura mater, skull and scalp. Intracranial interfaces, with contacts directly placed on brain tissues, ensure a much higher localization accuracy and signal-to-noise ratio. Electrocorticography (ECoG), either epidural or subdural, is an invasive approach widely used in seizure detection and functional mapping via electrode arrays placed mostly on the surface of the cerebral cortex and sometimes subcortical areas. Conventional ECoG uses macro-electrodes with a diameter of 1–4 mm and inter-contact space of 5–10 mm to record local field potentials. High-frequency neural activities that cannot be recorded using scalp EEG are of special interest for their close relation to cognitive functions. Recently introduced micro-ECoG electrodes with diameters ranging from 10 to 300 μm have demonstrated superior spatial resolution with less invasiveness [24]. Various ECoG-based BMI settings are proposed depending on the electrode distribution in non-epileptogenic areas [25], including motor control and imagination, speech, visual spelling, auditory and memory paradigms [26]. Direct electrical stimulation can be delivered via ECoG electrodes, enabling the bidirectional BMI paradigm and providing new opportunities for the treatment of neurological disorders [27]. While the design of ECoG limits its ability to probe deep into the brain, similarly to EEG, advanced algorithms make ECoG able to detect some source signals from subcortical structures [19].

Both Microelectrode array (MEA) and Microelectrode recording (MER) insert high-impedance electrodes into brain tissue and can record spike trains and local field potentials. MEAs are usually placed on functional cortical areas for high-dimensional multi-unit recordings and decoding of human intentions and functional activities, laying the foundation for different BMIs such as robotic arm control, text typing, speech neuroprosthesis and sensory restoration [8,28]. While MEAs are mainly used for surface recording, microelectrode recordings can be obtained from the deep brain with stereotactic targeting. MER is capable of both single channel and multi-channel recordings, with the latter aiming to bring in a more precise delineation of structural boundaries and more accurate localization of subregions based on specific electrophysiological activity patterns. When connected to a stimulator, MER can supply monopolar or bipolar stimulation, allowing the functional mapping of different brain states.

Electrodes of the endovascular neural recording and stimulating system, when first introduced, were placed in the segments of middle and anterior cerebral artery to detect epileptic foci without opening of the dura mater in patients with epilepsy in the 1990s. Recent progress in this invasive approach has involved the development of self-expanding stent-electrode arrays similar to traditional cerebrovascular stents, which can expand inside the vessels. The electrodes are connected to a transmission system within the body. This transmission system can communicate wirelessly with external devices. Recent attempts have tried to utilize this technology for the restoration of motor control based on cortical signals. Although the endovascular system is not as widely applied compared to other methods and many limitations remain, it provides a different way to interact with deep brain structures [29].

A high quality of neural signal transmission with sufficient temporal and spatial resolutions from different cortical and deep brain areas, minimal invasiveness and relatively long recording and stimulation times are key issues for BMI development. DBS and stereotactic electroencephalography (sEEG) are the most widely used interface technologies in current clinical practice that satisfy these requirements. Thus, we make special emphasis on these two BMI technologies, discuss their systematic constitution and clinical applications in detail, and provide insights into their significant roles in scientific research and future developments.

## INTERFACING WITH THE DEEP BRAIN

Previous research on BMI has mainly focused on decoding the brain signal and using it to encode an external device. However, modulating the brain could be more important in DBMIs. This section reviews deep interface technologies and their applications.

### Interface technology

DBS is an electrical neuromodulation therapy that involves minimally invasive deep brain implantation for chronic applications. Current DBS systems typically contain two intracranial electrodes connected to an implantable pulse generator (IPG) through extended wires. A standard electrode contains four to eight cylindrical contacts that are ∼1.5 mm in length, 1.2 mm in diameter and 0.5/1.5 mm in inter-contact space. With preoperative MRI-based targeting and intraoperative electrophysiological testing, electrodes of the DBS are implanted into the targeted deep brain. Electrical stimulation is generated in the IPG and delivered through implanted electrodes, of which the applied electric field is shaped by contact selection, amplitude, frequency and pulse width.

Recent advances in DBS include optimization in the electrode, IPG and programming system design, together with development of sensing-enabled and MRI-compactable technology. With the introduction of directional electrodes, while the top and the bottom contacts are still cylindrical, each of the middle two contacts is split into three segments, making up eight contacts in total for a lead [30]. More diverse electric fields can be realized with segmented contacts. The IPG is mostly implanted in the infraclavicular subcutaneous pocket, which involves neck tissue tunneling and chest incision. A new IPG design has reduced its size for implantation in the skull (NCT03837314). In addition, the technology for controlling multiple independent currents enables the automatic adjustment of stimulating parameters according to impedance changes, providing more accurate therapeutic delivery with fewer side effects [31]. Various attempts in waveform design and temporal pattern selection have been made to the programming system. Conventionally, the waveform of stimulation, a function of the current or voltage with respect to time, is asymmetrical with a short-duration cathodic phase of stimulus and a long-duration anodic phase of recharge. Newly developed stimuli with symmetrical waveforms may lead to superior suppression of motor symptoms in Parkinson's disease and essential tremor, as both the cathodic and anodic phases contribute to neuromodulation efficacy [32]. The temporal pattern of DBS is also a complex setting. Computational models and algorithms are investigated to determine the stimulus frequency and contact combinations [33,34]. Pattern selection can be viewed under the scope of parameter space exploration, which we will discuss in `Closed-loop deep brain-machine interface' Section.

The development of sensing and MRI-compatible DBS is an important trend in DBMI technology to provide more options for clinical application and promote research on human brain network dynamics. Sensing-enabled IPGs have the ability to extract electrophysiological signals through implanted electrodes. The recorded signals are mainly local field potential (LFP), the summing electrical activity of neurons in the target region [35]. Chronic sensing allows biomarker identification for closed-loop neuromodulation, which will be discussed in `Closed-loop deep brain-machine interface' Section. Previous DBS systems have prevented patients from MRI scanning due to the risks of device heating, current induction, IPG dysfunction and magnetic field-induced device movement. Heating is a major safety challenge for MRI compatibility. The conducting wires of the DBS lead interact with the radio frequency fields in MRI, posing the risk of excessive heating at the lead tip [36]. Recent progress in MRI-compatible DBS systems now allows simultaneous MRI scanning and electrical stimulation [37], making it possible to reveal deep brain stimulation-induced effects on neural activity at whole-brain level. New designs in the lead structure are proposed to diminish radio frequency heating and increase the intrinsic safety of the device, such as a braided shield to alter the resonance behavior [38–40] or wire winding with varied diameters to increase the outflow area of the induced currents [41]. Temperature-sensitive MRI parameters can be leveraged to derive the temperature rise when the electrode artifacts are taken care of [42]. Other physical quantities that are associated with the induced radio frequency currents can also be used, such as the B1 maps [43]. Besides radio frequency heating, the strong static magnetic field of the MRI can result in magnetic saturation of electronic components [44]. As ferromagnetic materials may cause magnetic forces and torques, it is better to use less ferromagnetic materials in the fabrication [37]. Currently, the most clinically endurable DBS device can work with radio frequency parameters as high as 3.4 μT in B_1+rms_ with 1 hour of continuous scanning under a 3.0T full-body MRI scan (G106R, PINS medical). DBS can simultaneously work with an MRI scan [40], which satisfies most research and clinical needs.

DBS is a successful interface for the clinical treatment of many neurological and psychiatric disorders. However, highly focused targets and limited channel numbers restrict its capability to sense and modulate large brain networks. Stereotactic electroencephalography (sEEG) could overcome these limitations with distributed recording and stimulation. It is a high-dimensional intracranial monitoring approach of subcortical structures to help localize epileptogenic zones and establish a 3D epileptogenic network. The electrodes of sEEG are typically made of platinum/iridium, containing 4–18 cylindrical contacts with a diameter of ∼0.8–1 mm, length of 2 mm and inter-contact interval of 2–10 mm. In clinical practice, usually 8–15 electrodes are implanted per patient to record from multiple brain areas with up to >100 channels simultaneously for several days to a few weeks. This allows capturing ictal and pre-/inter-ictal neural activities [45]. sEEG is also adopted in the identification of biomarkers of epileptogenic zones, the delineation of functional brain areas and electrical stimulation. The signals acquired by leads of the sEEG are also LFPs. A broad range of oscillations in different frequency bands from delta (0.5–3.5 Hz) to high gamma (>80 Hz) are recorded, providing both localized and distributed information with high spatial and temporal resolution [46]. The dynamics of neural activity and brain connectivity derived from electrodes outside epileptogenic zones and networks are of great value for clinical and neuroscientific research.

With the increasing application of sEEG, many researchers have leveraged its advantages in simultaneous recording from bilateral cortical and subcortical areas to conduct studies in perception, cognition and behavior while recording directly from the human brain. Here, broad aspects are covered including visual and auditory perception, attention, navigation, reward and decision-making, and many more [47]. The dynamics of brain connectivity and sleeping using sEEG recording has also attracted adequate interest [48]. Another important research topic is the development of sEEG-based BMI systems. Besides visual, speech and motor BMIs, sEEG allows interfacing with subcortical areas and developing novel BMI systems with deep brain signals [45].

Recently, progress has been made in the optimization of sEEG electrode placement. In the standard frame-based process, a detailed preoperative plan is defined by the clinical team to decide on targeted recording areas, surgical entry points and trajectories of implantation based on the information from MRI, EEG, MEG or PET. The sEEG leads are implanted with stereotactic guidance. Intraoperative fluoroscopy and CT scan can be used to confirm the position of electrodes. Recently, frameless neuronavigation has become widely used for an easier, more flexible and suitable procedure. Implantation precision and surgery time could be improved by robotic systems that can support the sEEG lead implantation in frame-based or frameless settings.

sEEG can be applied together with other stereotactic procedures. As an example, sEEG-guided radiofrequency–thermocoagulation combines sEEG investigation with radio frequency lesioning directly through the electrodes. During the procedure, preoperative vascular imaging and frame-based robotic assistance are used. sEEG and radiofrequency lesioning are used to treat epileptogenic zones inaccessible for surgery, large epileptic networks and periventricular nodular heterotopia [49].

### Clinical applications

DBS was first used in the alleviation of pain and psychiatric diseases including depression and anorexia [50]. In the 1970s, DBS was introduced for the treatment of movement disorders [51]. With the success of treating essential tremor by Benabid *et al.* [52,53], DBS entered the modern era. Today, DBS is a standard treatment for movement disorders including Parkinson's disease, essential tremor and dystonia, among which PD is the most common indication for DBS. Besides movement disorders, other neurological disorders such as epilepsy and Alzheimer's disease have become the new frontiers for DBS applications in recent years [54]. In addition, obsessive–compulsive disorder, Tourette syndrome, major depression disorders, addiction, anorexia nervosa and other psychiatric disorders are potential indications for DBS [55].

One major application of sEEG is to define the epileptogenic network in the brain. The direct approach is to record electrophysiological changes of different brain areas during ictal and interictal periods, thus mapping the level of seizures and propagation, and identifying distributed regions involved in seizures. Functional connectivity can also be analysed using linear or non-linear approaches measuring the correlation in the temporal and frequency domains of different regions. In addition, graph theory-based analysis allows discussion about both local and global features of epileptogenic networks [45]. sEEG is used as a functional mapping interface to identify the function of brain areas and assess susceptibility to stimulation-triggered epileptic seizures. The paradigm of stimulation includes low-frequency stimulation that targets areas of the lower after-discharge threshold and high-frequency stimulation [56]. sEEG could also be used in the evaluation of comorbidities of patients with epilepsy and guidance of subsequent interventional procedures [57]. First applications in this context have reported the localization of long-term modulation targets for treatment-resistant depression [58,59]. In the future, sEEG holds great potential in neurophysiological research and personalized treatment for other neurological and psychiatric disorders. Table 1 presents clinical applications for DBS and sEEG [2,54,60–67].

**Table 1.** Clinical applications for DBS and sEEG [2,54,60–67].

Disease	Targets
Parkinson's disease	Subthalamic nucleus, globus pallidus internus, ventral intermediate thalamic nucleus, pedunculopontine nucleus
Dystonia	Globus pallidus internus, subthalamic nucleus, ventral intermediate thalamic nucleus
Essential tremor	Ventral intermediate thalamic nucleus, posterior subthalamic area/caudal zona incerta
Multiple sclerosis tremor	Ventral intermediate thalamic nucleus, ventralis oralis anterior/ventralis oralis posterior thalamic nuclei
Depression	Subcallosal cingulate gyrus, nucleus accumbens, ventral capsule/ventral striatum, medial forebrain bundle, lateral habenula, inferior thalamic peduncle
Obsessive–compulsive disorder	Subthalamic nucleus, nucleus accumbens, ventral capsule/ventral striatum, nucleus accumbens
Tourette syndrome	Anterior limb of internal capsule, centromedian–parafascicular complex, globus pallidus externus, nucleus accumbens
Epilepsy ^a^	The anterior nucleus of the thalamus, hippocampus, centromedian nucleus of the thalamus, cerebellum, nucleus accumbens
Drug addiction	Nucleus accumbens
Huntington's disease	Globus pallidus internus, globus pallidus externus
Chronic pain	Sensory thalamus, the periaqueductal gray/periventricular gray
Conscious disturbance	Centromedian–parafascicular nuclei of thalamus, cuneiform nucleus, pallidum
Anorexia	Subcallosal cingulate, nucleus accumbens
Bipolar disorder	Subcallosal cingulate, nucleus accumbens, ventral capsule/ventral striatum
Alzheimer's disease	Fornix, ventral capsule/ventral striatum

^a^Indication for sEEG.

## DBMI AS A RESEARCH PLATFORM FOR BRAIN CONNECTIVITY

One important function of the DBMI is understanding deep brain activities, especially pathological states. Prior knowledge of neuroanatomy and neuropathophysiology has provided guidance for deep brain sensing and interpretation. Advances in brain–machine interface and artificial intelligence technologies could capture dynamic brain network activities [68,69]. The technology of MRI-compatible DBMI provides a platform for directly exploring changes in brain connectivity before and after modulation [70]. The emergence of sensing techniques in DBMIs and the joint recording of electrophysiological signals from multiple sites have also improved the temporal resolution of brain network dynamics. These studies provide more knowledge for neural network modulation and help us deepen the understanding of disease mechanisms, which will lead to the optimization of biomarkers, implantation targets and modulation patterns, towards the design of next-generation closed-loop systems.

Diffusion MRI (dMRI) and functional MRI (fMRI) are two popular techniques for modeling the structural tractography and functional connectivity of the active human brain [71]. Combined with DBS and advanced computational methods, dMRI and fMRI can reveal the impact of DBS on connectivity patterns of the human brain. High-quality normative connectivity data generated from diffusion weighted imaging or resting-state fMRI from healthy subjects have been used to investigate brain connectivity patterns related to DBS effects. Based on the normative connectome, for structural connectivity, the volume of tissue activated (VTA) by DBS is used as a seed to generate the probabilistic tractography map, while for functional connectivity, temporal correlation analysis among voxels sampled from VTA and every other voxel in the brain is conducted. Then various statistical methods and machine-learning algorithms can be used to evaluate the relevance and predictability of specific connections with clinical outcomes, leading to the identification of optimal target networks for neuromodulation [72]. Such research paradigms have been used in various pathological conditions including Parkinson's disease [73–75], essential tremor [76], dystonia, Tourette syndrome [77], obsessive–compulsive disorder, epilepsy, treatment-resistant depression and Alzheimer's disease, leading to new research avenues in the hope to optimize pre-surgical targeting and post-surgical modulation.

Due to safety considerations, previous attempts of simultaneous MRI scanning and DBS have been made mostly with 1.5T MRI. With current advances in MRI-compatible DBS, the stimulation can work with 3T MRI. A paradigm of On/Off stimulation combined with various frequency or electrode configurations has been conducted during MRI scanning, showing intriguing findings. Frequency-dependent activation of the GPi–thalamus–cerebellar circuit and deactivation of the M1–putamen–cerebellum were shown, which correlated with motor improvement in long-term observations [40]. The activation levels of monopolar and bipolar stimulation are quite different in the same patient [78]. These results demonstrate the possibility of precise modulation at an individual level via DBS.

Similar trends exist in sEEG-based research. When combined with fMRI, they complement each other in temporal and spatial resolutions. As the functional localization of sEEG at the individual level is the gold standard, the feasibility of non-invasive preoperative surgical planning using fMRI has been demonstrated by comparing fMRI and sEEG mappings of brain functional areas. On the other hand, sEEG can provide prior knowledge for the whole-brain analysis of fMRI. For example, local epileptic foci identified by sEEG can be combined with fMRI to study the whole brain and subcortical epileptic brain network. However, due to technical limitations, no simultaneous sEEG–fMRI study has been reported yet. Considering the corresponding characteristics of these two techniques, building an MRI-compatible sEEG system can help realize the high-resolution recording of both spatial and temporal signals, allow correlation analysis and causal inference among different structures, establish probabilistic dynamic connectivity maps under diverse states or tasks and lead to neural information acquisition and processing from local neural populations to the whole brain.

## CLOSED-LOOP DEEP BRAIN–MACHINE INTERFACE

Closing the sensory-control loop is a grand challenge for brain–machine interface research. DBS and sEEG, as the most prevalent clinical DBMIs, provide safe and chronic interfaces for decoding and modulating neural activities. These systems have been developed for the treatment of pathological brain activities underlying neurological and psychiatric disorders. The standard clinical protocols for using these systems are open-loop, the stimulation of which does not respond to disease-related biomarkers. As DBS and sEEG systems become more widely used in clinical treatments, the potential for closed-loop applications becomes clearer. Real-time efficacy improvement and reduction of side effects may be reached with closed-loop neurostimulation. The fact that the neural target population may adapt to chronic stimulation, e.g. through neuroplastic changes, calls for the temporal adjustment of neuromodulation in treatment. Closing the sensing-modulation loop of DBMIs could regulate brain activities based on temporal feedback.

### Current system and applications

Typically, a closed-loop DBMI system comprises three modules: (i) input—a sensing module measuring internal/external disease-related biomarkers; (ii) output—a stimulation module delivering stimulation patterns to modulate deep brain activities; (iii) control—an algorithmic module mapping input sensing signals to output stimulations. Development of a simultaneous sensing and stimulating technique is the first step in building closed-loop DBMI systems.

As mentioned above, various DBS systems with stimulation and sensing capabilities have been developed in recent years. Early studies of closed-loop modulation were carried out with external IPG [79,80]. Most of the closed-loop modulation was in PD patients, where local field potentials from the subthalamus nucleus with a defined threshold was used as the biomarker for bradykinesia and rigidity, and the stimulation amplitude was automatically adjusted. Such a paradigm was compared with conventional treatment and showed more efficacy and fewer side effects [79,81]. However, given the experimental settings of these studies, further evidence is required before large-scale clinical application. Recent advances include the design of sensing and modulating systems for longitudinal brain signal recording [82,83] and their potential to serve as the decoding and modulating platform for human cognitive and motion states [84]. A recent study showed five patients implanted with sensing-enabled DBS who achieved long-term wireless recording and adaptive stimulation for ≤15 months [85]. In addition, the initial outcome of a preliminary cohort with bilateral dual target bidirectional DBS (Summit RC + S) in PD patients has been reported to show long-term efficacy of adaptive DBS with dual targets [86].

Another applicable closed-loop DBMI system is the responsive neurostimulation (RNS) device, which was approved for the treatment of partial-onset medication refractory epilepsy in 2013 [87]. It monitors neural activities at the epileptic foci or through an electrocorticography strip on the brain surface continuously, and delivers therapeutic stimulation when a seizure onset is detected [88]. With increasing use of the RNS system, long-term data including treatment verification in medication refractory epilepsy have been accumulated, leading to more accurate prediction, personalized stimulation and effective network identification. Besides epilepsy, recent studies have demonstrated closed-loop modulation for treatment-resistant major depression [58], leading to the expansion of indications.

**Figure 4.**
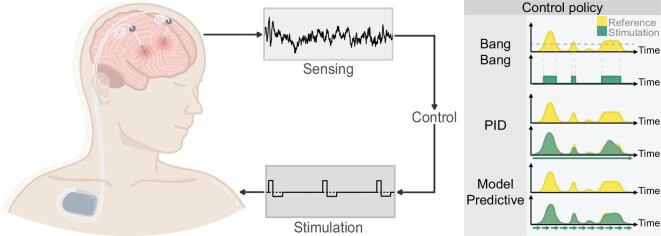
Sensing and modulation via a deep brain–machine interface. The implanted deep brain stimulator can record LFP signals and apply stimulation based on the sensing signals and the control policy. Control policies for current closed-loop deep brain stimulation can be categorized as Bang-Bang control, PID control and model predictive control.

Challenges for closed-loop DBMIs reside in three parts. The first part is sensing and decoding deep brain signals, where biomarker identification is the major challenge. The second part is encoding and stimulation, of which the main focus is stimulation parameter optimization. The last part is control, on how to stimulate properly based on the decoded signals (Fig. 4).

### Sensing and decoding

The sensing techniques of DBMIs enable recording of neural activities under various circumstances. Biomarkers in a closed-loop DBMI can be divided into disease-specific and state-related categories. The oscillation rhythms in LFP are the most frequently analysed biomarkers. The frequency-specific oscillations have been used to identify pathological states in patients. The most commonly used is beta band (13–35 Hz) power in the subthalamic nucleus (STN) in people with Parkinson's disease, which was shown to be a concrete biomarker for brady-rigidity [89], and showed long-term validity in follow-up studies [84,89]. Other oscillation features include increased theta band (4–8 Hz) power in STN, which is relevant to impulse control disorders [90]. Increased theta band activity in the GPi nucleus and centromedian–parafascicular thalamus indicate motor tics in Tourette syndrome [91] and high beta and gamma oscillations in GPi were considered to resist tics in the syndrome [92]. Besides these pathological biomarkers, state-related biomarkers have also been investigated. The LFP delta band recorded from STN in the full sleep state was more significant in the non-REM sleep stage than the awake stage, while the beta band was more significant in the awake stage and the REM sleep stage, with strong individual heterogeneity [93]. Some other studies attempted to establish sleep staging models based on STN local field potentials [94,95], which provided a promising basis for state-related modulation. A more recent study decoded the initiation, termination and vigor of leg muscle activation from STN LFPs and predicted the occurrence of freezing of gait in patients with PD [96], which might be applied to motor intention-based stimulation.

In addition to the frequency domain, the phase of oscillations also plays an important role. Oscillation biomarkers from local field potentials enable phase-lock encoding, which could provide a more accurate and comprehensive presentation of the disease states [97]. With the development of multi-site recording techniques, more network-related features have been investigated. Phase–amplitude coupling of beta and gamma bands is found in the primary motor cortex of patients with PD in the ‘OFF’ state [98,99]. Phase–amplitude coupling has also been found in patients with freezing of gait [100]. A recent study illustrated cortical response features evoked by DBS that are closely related to the hyperdirect pathway through which STN receives input signals from cortical regions [101]. Another study used intraoperative H-reflex measurement as a potential biomarker for optimal electrode positioning [102]. Some closed-loop systems could also take multi-modal signals such as acceleration and heart rate as biomarkers [103]. The tremor-phase tracked strategy can be used to control essential tremor with thalamus DBS [104]. These attempts show the progress in deep brain decoding for a closed-loop DBMI.

### Encoding and stimulation

Stimulation patterns include waveform and electrical field configuration. We have discussed the former previously (`Interfacing with the deep brain' Section). Here, we mainly discuss the electrical field configuration, which can be manually adjusted during programming. Several parameters influence the electrical field configuration: contact and polarity selection, amplitude, frequency and pulse width, among which frequency, or the temporal pattern, is closely related to neural oscillation and pathophysiology. Current DBS parameters are mostly set at constant frequencies. For instance, a commonly used frequency in DBS for PD is 130 Hz, which can alleviate symptoms of parkinsonism obviously. However, it may introduce deterioration of axial symptoms including gait, balance and speech disorders in the long run. Variable frequency stimulation was invented to balance the tradeoff of treatment effects. It can switch between different frequency patterns with selected intervals [105]. Studies have shown that variable frequency stimulation can increase gait speed, reduce the number of freezing episodes while alleviating symptoms of parkinsonism in patients with PD [106,107].

Another attempt at stimulation frequency optimization has been made on irregular stimulation based on the hypothesis that the temporal pattern, the precise timing of stimulation pulse sequence, plays an important role in neural coding [108]. Such a perception brings parameter settings into a huge space, making traditional iterative adjustment by physicians impossible. Computational models as well as machine-learning algorithms are applied in this field. A genetic algorithm, a sequential optimization method derived from the biological evolution principles, can be used in the highly complex optimization of non-linear systems [109]. For temporal pattern exploration, a genetic algorithm is used with computational models to predict the best non-regular pulse sequence in controlling parkinsonism [33].

Besides frequency modulation, more general exploration of parameter optimization has been conducted. As directional leads for DBS become more and more popular, the difficulty in programming stimulation parameters has increased significantly. With the help of MRI-compatible technology, the position of each electrode contact in the brain can be accurately mapped [70]. In addition, machine-learning algorithms have been applied to analyse clinical information for stimulation parameter optimization. Supervised learning algorithms such as random forest, support vector machines, Naïve Bayes and deep neural networks are widely used to retrospectively learn stimulation parameters and medication dosages for patients based on clinical ratings [110]. With sensing and imaging techniques, electrophysiological and imaging analyses are used to tune stimulation parameters with machine-learning methods. Applying support vector machines to a STN–LFP signal found the optimal contact of stimulation with an accuracy of 91% in a previous study with patient data [111]. Linear discriminant analysis was used to establish a learning model based on fMRI screening in Parkinson’s disease patients with optimal and non-optimal DBS settings, and predicted the optimal settings of contacts and amplitudes in unseen data sets [112].

Sequential optimization algorithms such as Bayesian optimization, genetic algorithm and simulated annealing have recently been used in neuromodulation pattern selection, and are similar to the process in which clinicians sequentially choose stimulation parameters and determine the optimal ones based on patient feedback. Bayesian optimization is a sequential search framework balancing between exploration and exploitation, and is often more efficient than grid search and random search [113] given the appropriate prior information in multidimensional spaces. In these studies, Bayesian optimization has been used in neuromodulation to find the optimal stimulation parameters based on personal preference [114,115] and electrophysiological recording [116,117]. In addition, combined with the finite element model of the human brain, a framework of simulating annealing was also established to optimize the electrical configuration, target and dosage of tDCS [118]. Safety is a top priority in the process of parameter optimization. New methods were developed to safely explore parameter space while guaranteeing optimization efficiency [119]. These investigations provide a broad prospect for DBMI encoding with advanced computational techniques.

### Control policies

When the scenario moves to a temporal dynamic closed-loop system, the feedback control strategy based on biomarkers is of great significance as it determines the following stimulation patterns given the patients’ pathological state estimation [120]. From the perspective of control theory, current feedback control policies used in a closed-loop DBMI can be divided into three categories: Bang-Bang controller, proportional integral derivative (PID) controller and model predictive controller (MPC).

Bang-Bang control, also referred to as responsive control or On/Off control, is a commonly used controller in closed-loop systems. Under Bang-Bang control, stimulation of a DBMI is delivered when the threshold value or pathological state is detected in real time. Such a strategy has been put into practice and has shown a promising reduction in stimulation time and alleviation of pathological symptoms. For instance, the threshold of the LFP beta band power in the STN was predefined in PD and intermittent stimulation was delivered when the threshold was reached [80,81]. Similar patterns have also been used in epilepsy treatment using the RNS system, which showed improvements in seizure reduction over time [121,122].

As Bang-Bang control may not satisfy the continuous and dynamic nature of some pathological states, PID control is taken into account. PID control monitors the error value continuously, and then calculates and combines the proportional, integral and derivative values together to create the output. In closed-loop DBMIs, PID simulation has been used in Parkinson's disease, showing higher efficiency and utility than the conventional pattern [123]. PID control has also been extended to application in neuroprosthesis for standing balance with functional electrical stimulation [124].

Model predictive control is a more advanced control policy. Due to the heterogeneity of neurological and psychiatric diseases and the existence of individual difference, patient-specific treatment or individual therapy is needed in complex situations, especially in a long-term closed-loop system. Therefore, apart from two control strategies mentioned above, model predictive control has received increasing attention recently. An MPC strategy was demonstrated based on the identified patient-specific symptoms response to DBS, leading to the optimization of closed-loop tremor control [125]. Another study constructed a multi-input multi-output state-delayed system that took the effect of time delay into consideration and showed higher efficiency than the conventional control policy based on simulation. Current applications of MPC are limited to simulation, so further studies will be needed to confirm the role of MPC in personalized neuromodulation.

## CONCLUSIONS AND FUTURE DIRECTIONS

Deep brain structures are essential to our vital functions including sensory, motor, cognition and consciousness. For the treatment of many neurological and psychiatric disorders, interacting with deep brain structures is unavoidable; therefore, deep brain–machine interfaces provide exclusive tools for directly recording deep brain activities, studying deep brain functions and networks, and modulating pathological deep brain states. The clinically applicable DBS and sEEG-based DBMIs enable additional therapeutic options for many diseases. While we still face big challenges in long-term safety, interaction efficacy, accessibility and other public concerns in neuroethics and regulations, with future development of novel interfacing technologies and advanced control methods, DBMIs would benefit more disabled people and neurological patients, and promote our understanding of neural networks and brain functions.
